# Role of gut microbiome in cancer immunotherapy: from predictive biomarker to therapeutic target

**DOI:** 10.1186/s40164-023-00442-x

**Published:** 2023-09-28

**Authors:** Mengwei Zhang, Jinkai Liu, Qiang Xia

**Affiliations:** 1 Department of Liver Surgery, Renji Hospital, Shanghai Jiao Tong University School of Medicine, Shanghai, China; 2 Shanghai Engineering Research Center of Transplantation and Immunology, Shanghai, China; 3 Shanghai Institute of Transplantation, Shanghai, China

**Keywords:** Gut microbiome, Cancer immunotherapy, Immunotherapy biomarkers, Immune checkpoint inhibitor, Fecal microbiota transplantation, Antibiotics

## Abstract

Immunotherapy has emerged as an effective treatment for various types of cancers. Recent studies have highlighted a significant correlation between the gut microbiome and patients’ response to immunotherapy. Several characteristics of the gut microbiome, such as community structures, taxonomic compositions, and molecular functions, have been identified as crucial biomarkers for predicting immunotherapy response and immune-related adverse events (irAEs). Unlike other -omics, the gut microbiome can serve as not only biomarkers but also potential targets for enhancing the efficacy of immunotherapy. Approaches for modulating the gut microbiome include probiotics/prebiotics supplementation, dietary interventions, fecal microbiota transplantation (FMT), and antibiotic administration. This review primarily focuses on elucidating the potential role of the gut microbiome in predicting the response to cancer immunotherapy and improving its efficacy. Notably, we explore reasons behind inconsistent findings observed in different studies, and highlight the underlying benefits of antibiotics in liver cancer immunotherapy.

## Introduction

Immunotherapy is a novel biotherapy designed to enhance immune responses against cancer [1]. Various immunotherapy drugs have been developed and employed in clinical trials or practice for cancer treatment [2]. Immune checkpoint inhibitors (ICIs), a class of drugs that target immune checkpoint molecules, are mostly used and show remarkable efficacy in several types of cancer [3, 4]. It is estimated that 1,290,156 patients are eligible for ICIs in China annually [5]. Despite the promising efficacy of immunotherapy, only a limited proportion of patients can benefit from it. The response rate was around 20% for liver cancer and melanoma patients [4, 6–9], and it only increased to approximately 30–50% in combination therapy [10–15]. Hence, it is imperative to efficiently identify predictive biomarkers linked to clinical response to immunotherapy. Although several immunotherapy biomarkers, such as expression level of programmed cell death ligand 1 (PD-L1), tumor mutational burden (TMB), and tumor-infiltrating T cells [16–20], were identified in different types of cancer, none of them was validated clinically. Additionally, these potential biomarkers are often intrinsic features that are challenging to manipulate, further limiting their practical application. Gut microbiome, the entire community of gastrointestinal (GI) microorganisms along with their genome and living environment [21], has recently been appreciated as an essential factor in immunotherapy [22–24]. Researchers proposed that gut microbiome can be used as both biomarkers and manipulating targets to predict and enhance the antitumor immunotherapy efficacy in different types of cancer [3, 25–27], which is critical for the precise application of immunotherapy and provides important guidance to patients' screening, precision tailoring, and response improving. This review provided a comprehensive overview of the gut microbiome's role in cancer immunotherapy, from response prediction to efficacy enhancement. To be specific, the practical characteristics of gut microbiome were categorized into the community structure, the taxonomic differences, and the functional molecular/pathway changes, and the manipulations of gut microbiome were summarized as overall and individual regulation including probiotics/prebiotics/dietary fibers supplementation, fecal microbiota transplantation (FMT), and antibiotics usage. In particular, we emphasized the dual function of antibiotics in cancer immunotherapy. Finally, we discussed the future directions for the application of gut bacteria in immunotherapy.

## The mechanisms of the influence of gut microbiome on immunotherapy

**Fig. 1:**
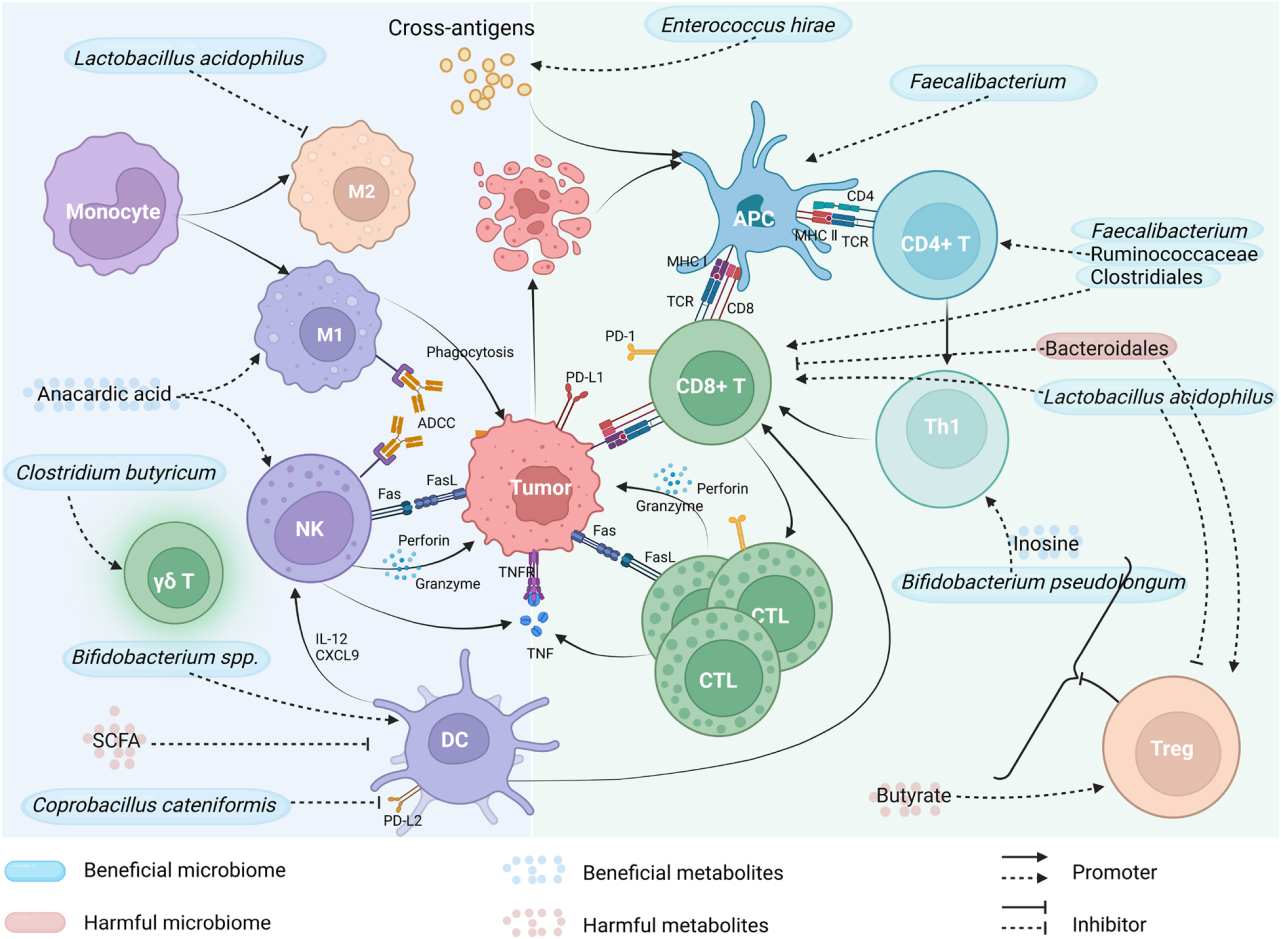
The mechanisms underlying the impact of gut microbiota and their metabolites on immunotherapy. *NK* natural killer, *DC* dendritic cell, *CTL* cytotoxic T lymphocyte, *APC* antigen-presenting cell, *Treg* regulatory T cell (created with BioRender.com)

The gut microbiome influences the effectiveness of immunotherapy mainly through regulating the immune system. Both innate and adaptive immunity could be regulated by the gut microbiome and their metabolites (Fig. 1) [28]. A higher density of immune cells and antigen processing/presentation markers were found in patients with high enrichment of *Faecalibacterium* [29]. A recent study found that the gut microbiome promotes antitumor immunity by suppressing the expression of PD-L2 and its binding partner repulsive guidance molecule b (RGMb). The mediator responsible for this effect was identified as *Coprobacillus cateniformis*, which was found to downregulate PD-L2 expression on dendritic cells (DCs) and increase the efficacy of programmed cell death protein 1 (PD-1) inhibitors [30]. The *Faecalibacterium*, Ruminococcaceae, and Clostridiales were enriched in PD-1 inhibitor responders of melanoma and showed a significantly positive correlation with CD8^+^ T cell infiltrate, as well as frequencies of effector CD4^+^ and CD8^+^ T cells in the circulation [29]. Meanwhile, Bacteroidales, which are associated with non-responders, showed correlations with reduced infiltration of CD8^+^ T cells in tumors and elevated levels of regulatory T cells (Tregs) and myeloid-derived suppressor cells (MDSCs) in the circulation [29]. Recently, a study has revealed that melanoma patients treated with combined ICIs and developed immune-related adverse events (irAEs) of grade three or higher exhibited an increased abundance of *Bacteroides intestinalis*. The research suggests that *B. intestinalis* may trigger the occurrence of irAEs by inducing ileal IL-β1 expression, which can be prevented by administering an IL-1R antagonist [31].

The cross-reaction between microbial antigens and tumor antigens has been found important for the antitumor effect of gut microbiome [32]. Therefore, the microbiome components may enhance the efficacy of immunotherapy to a certain degree. Vétizou et al. found that specific Bacteroides species are crucial for the antitumor effects of CTLA-4 blockade. Oral administration of either *Bacteroides thetaiotaomicron* or *B. fragilis* to antibiotic-treated or germ-free mice was sufficient to recover these effects [33]. Further analysis revealed that not only the gavage with *B. fragilis* but also immunization with *B. fragilis* polysaccharides can rescue the deficiency of response to CTLA blockade observed in antibiotic-treated or germ-free mice, and a similar effect was also observed upon adoptive transfer of *B. fragilis*-specific T cells [33], highlighting the role of microbiota-associated immune reactions rather than live microorganisms in enhancing immunotherapy. Similarly, Zhuo et al. combined *Lactobacillus acidophilus* lysates with CTLA-4 blockade to treat BALB/c mice models of colorectal cancer (CRC). The combination therapy enhanced the antitumor effect of CTLA-4 blockade, resulting in slower weight loss and fewer tumors by increasing CD8^+^ T cells and memory T cells while decreasing immunosuppressive cells such as Treg and M2 macrophages [34].

In addition to bacteria, other members of the gut microbiome may also serve as predictors for immunotherapy response. Fluckiger et al. found that a protein epitope from a prophage, which was present in the genome of bacteriophage *Enterococcus hirae*, exhibits cross-reactivity with tumor MHC class I-restricted antigens. *E. hirae* strains containing this epitope show antitumor effects and can elicit specific T cell responses during immunotherapy, while the absence or mutation of this epitope is associated with a lack of antitumor effects. Furthermore, the presence of this prophage in fecal specimens corresponded to enhanced efficacy of immunotherapy in patients with renal or lung cancers [32].

Short-chain fatty acids (SCFAs) are important metabolites produced by gut microbiota, which have the potential to modulate immune system. In a mouse model, it was found that SCFAs can limit the activity of anti-CTLA-4 by restricting the up-regulation of CD80/CD86 on DCs and ICOS on T cells, as well as the accumulation of tumor-specific and memory T cell [35]. Butyrate, a four-carbon SCFA, can induce the differentiation of Tregs in liver [36], which may suppress the antitumor immunity of immunotherapy. It is worth noting that the metabolic products might be correlated with specific bacteria strains, thus assessing their relationship is of great significance. For example, ursodeoxycholic acid (UDCA) and ursocholic acid (UCA) (enriched in responders) were significantly associated with the enrichment of *Lachnoclostridium* [37]. Positive correlations were also found between the anti-PD-1/PD-L1 response and the SCFA-producing gut bacteria (such as *Eubacterium*, *Lactobacillus*, and *Streptococcus*) in different GI cancers [27]. Inosine is a nucleoside that plays an important role in the metabolism of purines. It has been demonstrated that the production of the gut-derived inosine by intestinal *Bifidobacterium pseudolongum* resulted in an enhanced immunotherapy response through T cell expression of adenosine A_2A_ receptor and costimulation [38]. Anacardic acid, an alkyl derivative of salicylic acid mainly produced from the nutshell of cashews, was found to remarkably increase in responders, which can be explained by its ability to stimulate neutrophils/macrophages and enhance T-cell recruitment, and consequently improve immunotherapy [28, 39].

Last but not least, the gut microbiome might be shaped by cancer immunotherapies. For example, when compared to healthy controls, the abundance of *Bacteroides plebeius*, *Lactobacillus*, *Prevotella*, *Streptococcus*, *Oscillospira*, Rikenellaceae, and Enterobacteriaceae was higher during Nivolumab treatment in NSCLC patients [40]. There are also studies comparing the changes in gut microbiome before and after immunotherapy. Little changes were observed in the relative abundance of the top 20 most abundant microbes in NSCLC patients before and during immunotherapy (at baseline, from 1 to 4 treatment cycles, and when disease progressed) [41]. However, the gut microbiome associated with immunotherapy was found altered in response to immunotherapy. As mentioned above, different Bacteroides species, such as *B. thetaiotaomicron* or *B. fragilis*, are required for the anticancer effects of CTLA-4 blockade in mice and humans. The abundance analysis of Bacteroidales and *Bacteroides* before and 2 weeks after immunotherapy showed that ipilimumab can facilitate the colonization of *B. thetaiotaomicron* or *B. fragilis* [33]. These results suggested the reciprocal influence between the gut microbiome and immunotherapy and highlighted the importance of studying the gut microbiome throughout the process of immunotherapy.

## Effectiveness prediction of immunotherapy

The gut microbiome is a stable and diverse part of the human body. The gut microbial community can be relatively stable in a certain period at the individual level, which indicates that there is a stable association between gut microbial status and individual health. Heterogeneity of gut microbiome across individuals could be resulted from confounding factors such as genetics, diet, environment, drugs, and smoking. Previous research has identified these differences can be used to classify populations including drug responders and nonresponders populations [42–44]. As mentioned in the previous section, intense crosstalks have been discovered between gut microbes and the immune system [25, 29, 45]. The gut microbiome affects the development and function of the immune system in a variety of ways, such as regulating the differentiation of lymphocytes, natural killer (NK) cells, and Tregs [25, 29, 45]. This close connection provides a theoretical possibility for predicting the efficacy of immunotherapy. In addition, gut microbiome, represented by stool samples, could be easily acquired, which enables clinicians to obtain baseline pre-immunotherapy microbiome data. In fact, there have been many studies that identified the connection between gut microbiome characteristics and immunotherapy efficacy, and the baseline gut microbiome information is recognized as a suitable candidate for predicting the response to immunotherapy [46–49].

**Fig. 2:**
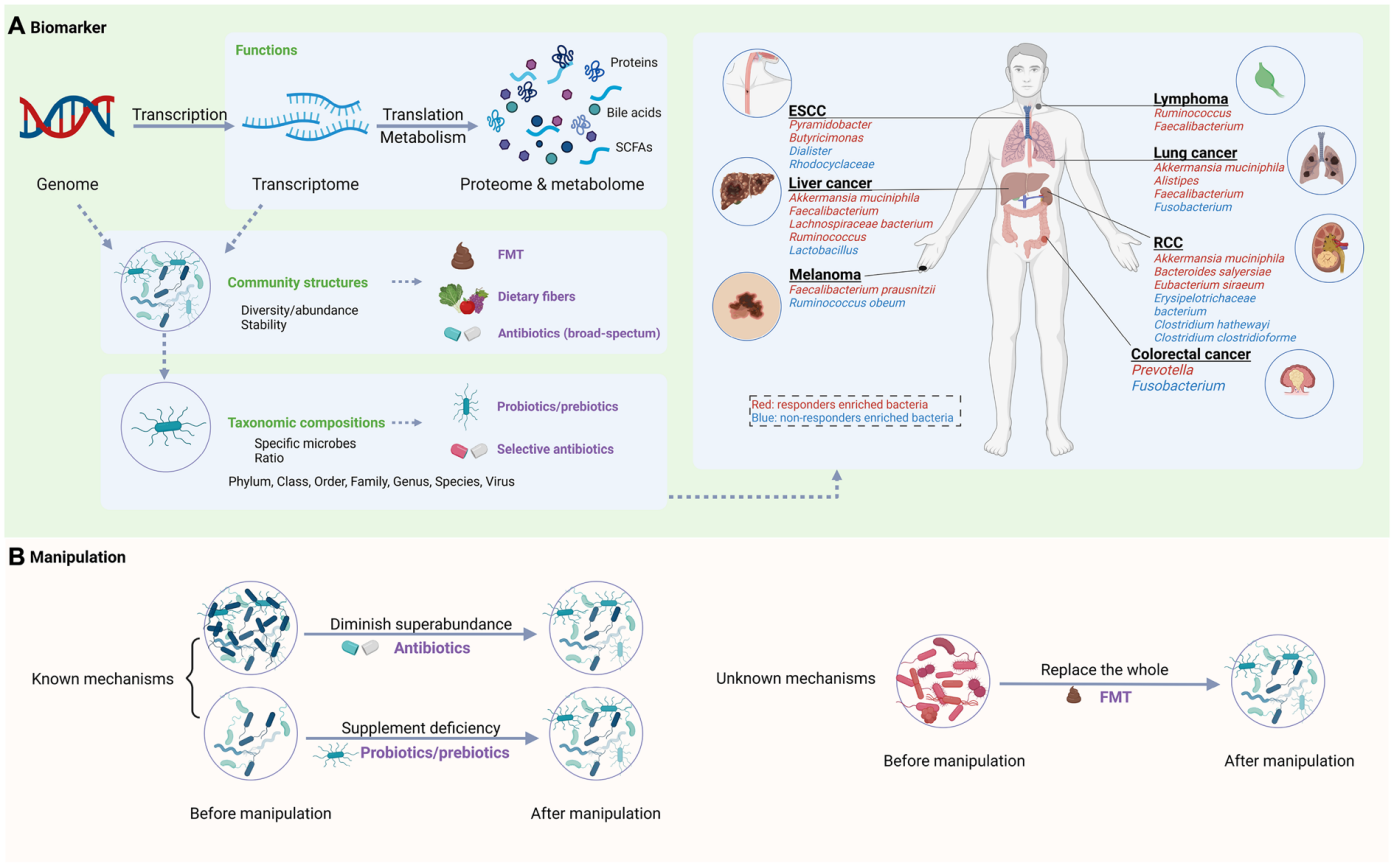
The role of gut microbiome in immunotherapy. **A** Gut microbiome biomarkers for immunotherapy. **B** Manipulation of the gut microbiome to enhance the efficiency of immunotherapy (created with BioRender.com)

The gut microbiome is a complex community with various features. To put it clearly, we classified the potential predictive characteristics of the gut microbiome into three categories: (i) the community structures, (ii) taxonomic compositions, and (iii) function factors (Fig. 2A). In short, community structures reflect the general characteristics of the microbiome, such as the diversity of the gut microbiome. The taxonomic composition refers to the specific microorganisms that can be manipulated easily and individually with great translational and controllable potential. The function factors include gene expression-related factors such as the metabolic pathways and protein/metabolic products, which may be more direct and accurate biomarkers due to their closest relationship with the mechanisms.

### Community structures

It is well established that the diversity or abundance of the gut microbiome is a possible biomarker for predicting the prognosis of diseases, including the prediction of immunotherapy response. Low microbiota diversity was observed in chronic diseases and related to poor prognosis in cancer therapy [29]. Similarly, patients who have gut microbes with lower diversity or species richness are less likely to respond to immunotherapy and experience shorter progression-free survival (PFS) [50–52]. Higher species richness or diversity has been found in responders with non-small-cell lung cancer (NSCLC), hepatocellular carcinoma (HCC), or melanoma at baseline compared with non-responders [29, 31, 41, 48, 53, 54]. Though most studies revealed positive associations, a few studies, especially studies with relatively small sample sizes, failed to testify significant differences between gut microbiota species abundances and response to immunotherapies. One possible reason for these heterogeneous results is the lack of sufficient patient samples, which may make random errors dominant.

Moreover, the high diversity of gut microbiome in responders remains stable during the immunotherapeutic process, which is different from the specific bacteria strains [41, 48, 55]. In a Chinese cohort with NSCLC, stool samples were collected at baseline and at eight consecutive time points (every 2 weeks) after immunotherapy. No significant changes were observed in Shannon diversity index or gut microbiota composition at the genus level among different time points. The authors also conducted PCoA analysis of gut microbiome in 10 patients (seven responders and three non-responders). Gut microbiome at different time points could not be divided into obvious clusters, while responders and non-responders were separated clearly [48]. Zhang et al. carried out a similar longitudinal sampling strategy to dynamically evaluate the gut microbiome in NSCLC patients throughout anti-PD-1 treatment. Five sampling time points were chosen from baseline to disease progressed. There was no significant difference in alpha or beta diversity at different time points [41]. These findings suggested that the community structures of gut microbiome remained largely stable throughout the immunotherapy, and this stability enables it as a stable biomarker for response prediction.

### Taxonomic differences

**Fig. 3:**
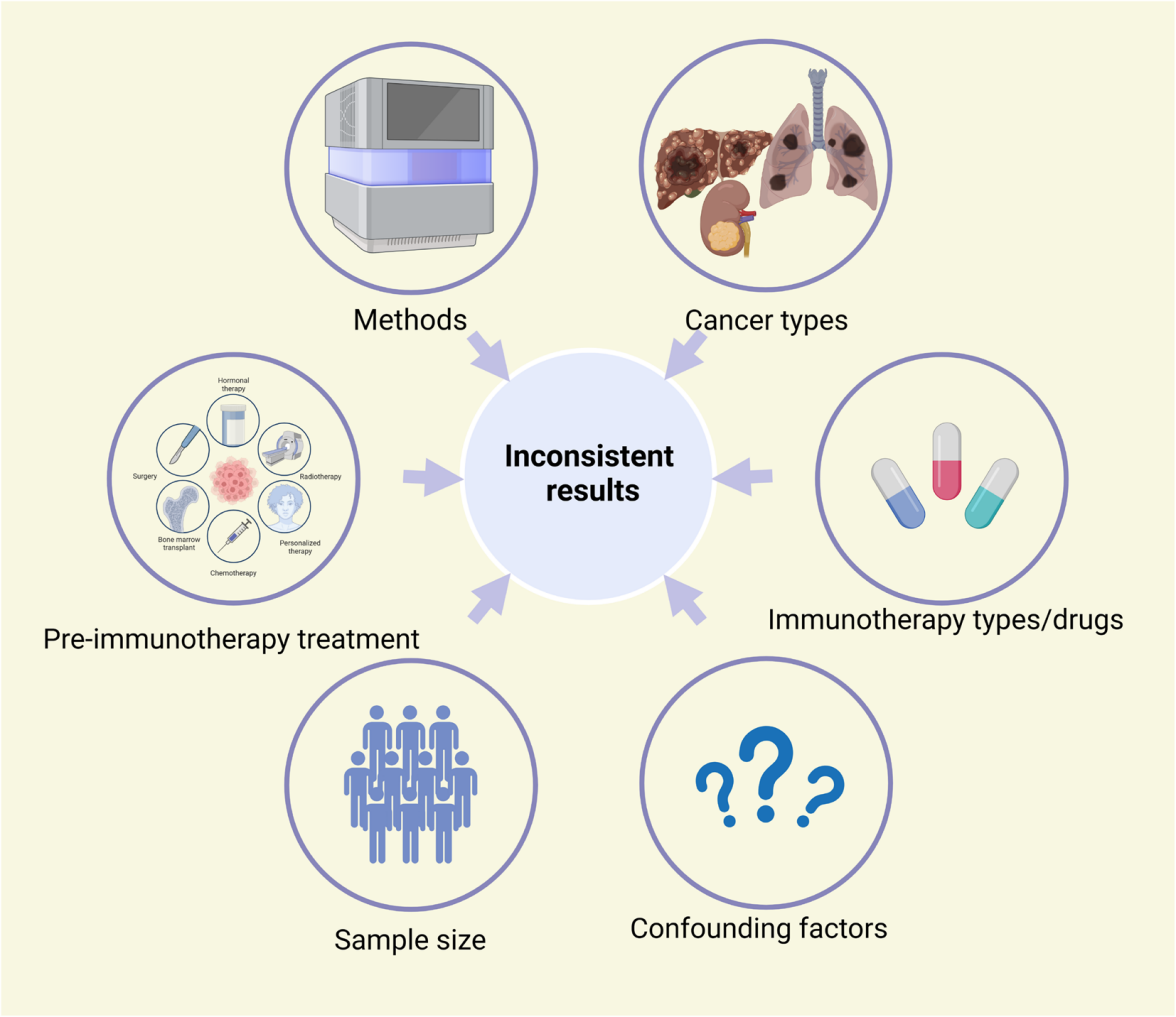
Causes of inconsistent results among different studies (created with BioRender.com)

The taxonomic composition of gut microbes (specific microorganisms) may serve as ideal markers for immunotherapy prognosis due to their accuracy in prediction tasks and convenience for clinical supplement or deletion. Specific differences in the microbiome composition were found in both responders and non-responders at different taxonomy levels (including phylum, class, order, family, genus, species, and even strains) (Tables 1, 2). Despite the promising results of the predictive role of the gut microbiome in cancer immunotherapy, consistent results have not been obtained, possibly due to the dynamic, complex, and susceptible nature of gut microbiome (Fig. 3).

**Table 1:** Potential gut microbiota enriched in responders

Cancers	Pre-treatment	Immunotherapy	Response criteria	Sequencing methods	Level	Bacteria	Sample size	Countries/territories	Year	References
HCC	–	Nivolumab or pembrolizumab	RECIST 1.1	16S rDNA (V3–V4 region)	Family	Lachnospiraceae	74	Taiwan	2022	[37]
HCC	None	Tremelimumab and/or Durvalumab	mRECIST	16 s rDNA (V3–V4 region)	Genus	*Akkermansia*	11	Italy	2022	[56]
HCC	–	Nivolumab or pembrolizumab	RECIST 1.1	16S rDNA (V3–V4 region)	Genus	*Lachnoclostridium* and *Veillonella*	74	Taiwan	2022	[37]
HCC	–	ICIs	RECIST 1.1	16S rDNA (V4 region)	Genus	*Faecalibacterium*	65	China	2020	[57]
HCC	–	Nivolumab	RECIST 1.1	16S rDNA (V3–V4 region)	Genus	*Akkermansia*	8	South Korea	2021	[53]
HCC	None	PD-1 inhibitors	mRECIST	16S rDNA (V3–V4 region)	Genus	*Faecalibacterium*, *Blautia*, *Megamonas*, *Ruminococcus*, *Coprococcus*, *Dorea*, and *Haemophilus*	35	China	2022	[54]
HCC	Sorafenib	PD-1 inhibitors	RECIST 1.1	Metagenomics	Species	*Akkermansia muciniphila* and *Ruminococcaceae* spp.	8	China	2019	[47]
HCC	–	Nivolumab	RECIST 1.1	16S rDNA (V3–V4 region)	Species	*Citrobacter freundii*, *Azospirillum* sp. and *Enterococcus durans*	8	South Korea	2021	[53]
HCC	None	PD-1 inhibitors	mRECIST	16S rDNA (V3–V4 region)	No rank	*Lachnospiraceae incertae Sedis*	35	China	2022	[54]
Hepatobiliary cancers	Chemotherapy (gemcitabine plus cisplatin)	PD-1 inhibitors	RECIST.1.1	Metagenomics	Species	*Lachnospiraceae bacterium*-GAM79 and *Alistipes* sp. *Marseille*-P5997	65	China	2021	[46]
Hepatobiliary cancers	Chemotherapy (gemcitabine plus cisplatin)	PD-1 inhibitors	RECIST.1.1	Metagenomics	Species	*Ruminococcus calidus* and *Erysipelotichaceae bacterium*-GAM147	65	China	2021	[46]
NSCLC	Platinum-based doublets chemotherapy	PD-1 inhibitors	RECIST 1.1	Metagenomics	Class	Clostridia, Bacteroidia	85	China	2022	[58]
NSCLC	Platinum-based chemotherapy	ICIs	RECIST 1.1	16S rDNA (V3–V4 region)	Order	Actinomycetales	75	China	2021	[41]
NSCLC	Platinum-based chemotherapy	ICIs	RECIST 1.1	16S rDNA (V3–V4 region)	Family	Odoribacteraceae and Rikenellaceae	75	China	2021	[41]
NSCLC	–	PD-1/PD-L1 inhibitors	–	16S rDNA (V3–V4 region)	Family	Akkermansiaceae	47	Poland	2022	[59]
NSCLC	–	PD-1/PD-L1 inhibitors	RECIST 1.1	16S rDNA (V3–V4 region)	Genus	*Ruminococcaceae* UCG 13 and *Agathobacter*	70	Japan	2020	[60]
NSCLC	–	PD-1 inhibitors	RECIST 1.1	Metagenomic	Genus	*Parabacteroides* and *Methanobrevibacter*	63	China	2020	[51]
NSCLC	Platinum-based chemotherapy	ICIs	RECIST 1.1	16S rDNA (V3–V4 region)	Genus	*Desulfovibrio*, *Bifidobacterium*, *Anaerostipes*, *Faecalibacterium*, and *Alistipes*	75	China	2021	[41]
NSCLC	Did not receive previous targeted therapy	ICIs	RECIST 1.1	16S rDNA (V3–V4 region)	Genus	*Phascolarctobacterium*	69	Spain	2021	[61]
NSCLC	–	ICIs	RECIST 1.1	16S rDNA (V1–V3 region)	Genus	*Ruminococcus*, *Akkermansia*, and *Faecalibacterium*	65	USA	2022	[62]
NSCLC	–	ICIs	RECIST 1.1	16S rDNA (V1–V2 region)	Genus	*Lactobacillus*, *Clostridium*, and *Syntrophococcus*	17	Japan	2019	[63]
NSCLC	At least one prior line of treatment	PD-1 inhibitors	RECIST 1.1	Metagenomics	Species	*Ruminococcus* spp., *Alistipes* spp., and *Eubacterium* spp.	60	France	2018	[3]
NSCLC	–	Nivolumab	RECIST 1.1	16S rDNA (V3–V4 region)	Species	*Alistipes putredinis*, *Bifidobacterium longum*, and *Prevotella copri*	37	China	2019	[48]
NSCLC	Platinum-based doublets chemotherapy	PD-1 inhibitors	RECIST 1.1	Metagenomics	Species	*Bacteroides massiliensis* (igc0097), *Alistipes obesi (igc0342)*, *Alistipes obesi, Akkermansia muciniphila*	85	China	2022	[58]
NSCLC (n = 60 + 27) and RCC (n = 40 + 26)	At least one prior line of treatment	PD-1 inhibitors	RECIST 1.1	Metagenomics	Species	*Akkermansia muciniphila*	100 + 53	France	2018	[3]
Lung cancer	–	Monotherapy or in combination with chemotherapy	–	16S rDNA (V3–V4 region)	Order	Clostridiales	34	USA	2021	[64]
Melanoma	–	Ipilimumab	–	16S rDNA (V3–V4 region)	Phylum	Firmicutes	26	France	2017	[25]
Melanoma	–	PD-1 inhibitors	RECIST 1.1	16S rDNA	Order	Clostridiales	43	USA	2018	[29]
Melanoma	–	PD-1 inhibitors	RECIST 1.1	16S rDNA	Family	Ruminococcaceae	43	USA	2018	[29]
Melanoma	–	Ipilimumab	–	16S rDNA (V3–V4 region)	Genus	*Faecalibacterium*	26	France	2017	[25]
Melanoma	–	PD-1 inhibitors	RECIST 1.1	16S rDNA and metagenomics	Genus	*Faecalibacterium*	43	USA	2018	[29]
Melanoma	–	ICIs	–	16S rDNA and metagenomics	Species	*Faecalibacterium prausnitzii*, *Coprococcus eutactus*, *Prevotella stercorea*, *Streptococcus sanguinis*, *Streptococcus anginosus*, and *Lachnospiraceae bacterium* 3 1 46FAA	27	USA	2019	[50]
Melanoma	–	ICIs	RECIST 1.1	16S rDNA (V4 region) and metagenomics	Species	*Bacteroides stercoris*, *Parabacteroides distasonis* and *Fournierella massiliensis*	54 and 38	USA	2021	[31]
Melanoma	None (72%)	Ipilimumab	–	16S rDNA	Species	*Bacteroides thetaiotaomicron* and *Bacteroides fragilis*	25	France; USA	2015	[33]
Melanoma	–	PD-1/CTLA-4 inhibitors	RECIST 1.1	16S rDNA (V4 region) and metagenomics	Species	*Bifidobacterium longum*, *Collinsella aerofaciens*, *Enterococcus faecium*, *Bifidobacterium adolescentis*, *Klebsiella pneumoniae, Veillonella parvula, Parabacteroides merdae*, and* Lactobacillus* sp.	42	USA	2018	[65]
Melanoma	–	Ipilimumab, Nivolumab, Ipilimumab plus Nivolumab, or pembrolizumab	RECIST 1.1	Metagenomics	Species	*Bacteroides caccae*	39	USA	2017	[39]
Melanoma	–	Ipilimumab plus Nivolumab	RECIST 1.1	Metagenomics	Species	*Faecalibacterium prausnitzii*, *Bacteroides thetaiotamicron*, and *Holdemania filiformis*	24	USA	2017	[39]
Melanoma	–	Pembrolizumab	RECIST 1.1	Metagenomics	Species	*Dorea formicogenerans*	13	USA	2017	[39]
GI cancer	–	PD-1/PD-L1 inhibitors	RECIST 1.1	16S rDNA (V3–V4 region)	Family	Ruminococcaceae and Lachnospiraceae	74	China	2020	[27]
GI cancer	–	PD-1/PD-L1 inhibitors	RECIST 1.1	16S rDNA (V3–V4 region)	Genus	*Prevotella*	74	China	2020	[27]
GI cancer	–	PD-1/PD-L1 inhibitors	RECIST 1.1	16S rDNA (V3–V4 region)	Ratio (genus)	Elevation of the Prevotella/Bacteroides ratio	74	China	2020	[27]
B cell malignancies	–	Anti-CD19 CAR T cell therapy	–	16S rDNA (V4–V5 region)	Class	Clostridia	48	USA	2022	[66]
B cell malignancies	–	Anti-CD19 CAR T cell therapy	–	16S rDNA (V4–V5 region)	Family	Ruminococcaceae	48	USA	2022	[66]
B cell malignancies	–	Anti-CD19 CAR T cell therapy	–	16S rDNA (V4–V5 region)	Genus	*Ruminococcus* and *Faecalibacterium*; *Bacteroides*	48	USA	2022	[66]
B cell malignancies	–	Anti-CD19 CAR T cell therapy	–	16S rDNA (V4–V5 region)	Species	*Faecalibacterium prausnitzii* and *Ruminococcus bromii*	48	USA	2022	[66]
ESCC	Neoadjuvant camrelizumab and chemotherapy	Camrelizumab plus carboplatin and paclitaxel before surgery	RECIST 1.1	16S rDNA (V3–V4 region)	Family	Barnesiellaceae, Dethiosulfovibrionaceae, Odoribacteraceae,	44	China	2022	[67]
ESCC	Neoadjuvant camrelizumab and chemotherapy	Camrelizumab plus carboplatin and paclitaxel before surgery	RECIST 1.1	16S rDNA (V3–V4 region)	Genus	*Pyramidobacter*, *Butyricimonas*, *Prevotella*, *Barnesiella*, and *Odoribacter*	44	China	2022	[67]
Thoracic carcinoma	–	PD-1 inhibitors	RECIST 1.1	16S rDNA (V4 region)	Family	Akkermansiaceae, Enterococcaceae, Enterobacteriaceae, Carnobacteriaceae and Clostridiales Family XI	42	China	2021	[68]
Renal and lung cancer patients	–	PD-1 inhibitors	–	–	Virus	Enterococcal prophage	–	–	2020	[32]
RCC	TKI (68%)	Nivolumab	RECIST 1.1	Metagenomics	Species	*Akkermansia muciniphila*, *Bacteroides salyersiae*, and *Eubacterium siraeum*, *Clostridium ramosum* (ns), *Alistipes senegalensis* (ns)	58	France	2020	[55]

*HCC* hepatocellular carcinoma, *NSCLC* non-small-cell lung cancer, *GI* gastrointestinal, *ESCC* esophageal squamous cell carcinoma, *RCC* renal cell carcinoma, *ICIs* immune checkpoint inhibitors, *RECIST* Response Evaluation Criteria in Solid Tumors, *PD-1* programmed death 1, *PD-L1* programmed death ligand 1

**Table 2:** Potential gut microbiota enriched in non-responders

Cancers	Pre-treatment	Immunotherapy	Response criteria	Sequencing methods	Level	Bacteria	Sample size	Countries/territories	Year	References
HCC	–	Nivolumab or pembrolizumab	RECIST 1.1	16S rDNA (V3–V4 region)	Genus	*Prevotella 9*	74	Taiwan	2022	[37]
HCC	–	ICIs	RECIST 1.1	16S rDNA (V4 region)	Genus	*Bacteroidales*	65	China	2020	[57]
HCC	None	PD-1 inhibitors	mRECIST	16S rDNA (V3–V4 region)	Genus	*Atopobium*, *Leptotrichia*, *Campylobacter*, *Allisonella*, *Methanobrevibacter*, *Parabacteroides*, *Bifidobacterium* and *Lactobacillus*	35	China	2022	[54]
HCC	–	Nivolumab	RECIST 1.1	16S rDNA (V3–V4 region)	Species	*Dialister pneumosintes, Escherichia coli, Lactobacillus reteri, Streptococcus mutans, Enterococcus faecium, Streptococcus gordonii, Veillonella atypica, Granulicatella* sp., and *Trchuris trichiura*	8	South Korea	2021	[53]
HCC	–	Nivolumab	RECIST 1.1	16S rDNA (V3–V4 region)	Ratio (genus)	Low *Prevotella/Bacteroides* ratio	8	South Korea	2021	[53]
HCC	–	Nivolumab	RECIST 1.1	16S rDNA (V3–V4 region)	Ratio (phylum)	Skewed Firmicutes/Bacteroidetes ratio	8	South Korea	2021	[53]
Hepatobiliary cancers	Chemotherapy (gemcitabine plus cisplatin)	PD-1 inhibitors	RECIST.1.1	Metagenomics	Family	Veillonellaceae	65	China	2021	[46]
NSCLC	Platinum-based chemotherapy	ICIs	RECIST 1.1	16S rDNA (V3–V4 region)	Phylum	Fusobacteria	75	China	2021	[41]
NSCLC	–	PD-1 inhibitors	RECIST 1.1	Metagenomic	Class	Negativicutes	63	China	2020	[51]
NSCLC	Platinum-based chemotherapy	ICIs	RECIST 1.1	16S rDNA (V3–V4 region)	Class	Fusobacteriia	75	China	2021	[41]
NSCLC	–	PD-1 inhibitors	RECIST 1.1	Metagenomic	Order	Selenomonadales	63	China	2020	[51]
NSCLC	Platinum-based chemotherapy	ICIs	RECIST 1.1	16S rDNA (V3–V4 region)	Order	Fusobacterales	75	China	2021	[41]
NSCLC	Platinum-based chemotherapy	ICIs	RECIST 1.1	16S rDNA (V3–V4 region)	Family	Fusobacteriaceae	75	China	2021	[41]
NSCLC	–	PD-1 inhibitors	RECIST 1.1	Metagenomic	Genus	*Veillonella*	63	China	2020	[51]
NSCLC	Platinum-based chemotherapy	ICIs	RECIST 1.1	16S rDNA (V3–V4 region)	Genus	*Fusobacterium*	75	China	2021	[41]
NSCLC	Did not receive previous targeted therapy	ICIs	RECIST 1.1	16S rDNA (V3–V4 region)	Genus	*Dialister*	69	Spain	2021	[61]
NSCLC	–	ICIs	RECIST 1.1	16S rDNA (V1–V2 region)	Genus	*Bilophila*, *Sutterella*, and *Parabacteroides*	17	Japan	2019	[63]
NSCLC	At least one prior line of treatment	PD-1 inhibitors	RECIST 1.1	Metagenomics	Species	*Bifidobacterium adolescentis*, *B. longum*, and *Parabacteroides distasonis*	60	France	2018	[3]
NSCLC	–	Nivolumab	RECIST 1.1	16S rDNA (V3–V4 region)	Species	*Ruminococcus_unclassified*	37	China	2019	[48]
NSCLC	Platinum-based doublets chemotherapy	PD-1 inhibitors	RECIST 1.1	Metagenomics	Species	*Bacteroides fragilis* (igc0079)	85	China	2022	[58]
Melanoma	–	PD-1 inhibitors	RECIST 1.1	16S rDNA	Order	Bacteroidales	43	USA	2018	[29]
Melanoma	–	Ipilimumab	–	16S rDNA (V3–V4 region)	Genus	*Bacteroides*	26	France	2017	[25]
Melanoma	–	ICIs	–	16S rDNA and metagenomics	Species	*Bacteroides ovatus*, *Bacteroides dorei*, *Bacteroides massiliensis*, *Ruminococcus gnavus*, and *Blautia producta*	27	USA	2019	[50]
Melanoma	–	ICIs	RECIST 1.1	16S rDNA (V4 region) and metagenomics	Species	*Klebsiella aerogenes* and *Lactobacillus rogosae*	54 and 38	USA	2021	[31]
Melanoma	–	PD-1 inhibitors	RECIST 1.1	Metagenomics	Species	*Bacteroides thetaiotaomicron*, *Escherichia coli*, and *Anaerotruncus colihominis*	43	USA	2018	[29]
Melanoma	–	PD-1/CTLA-4 inhibitors	RECIST 1.1	16S rDNA (V4 region) and metagenomics	Species	*Ruminococcus obeum* and *Roseburia intestinalis*	42	USA	2018	[65]
Colorectal cancer	Chemotherapy	Regorafenib plus toripalimab	RECIST 1.1	16S rDNA (V3–V4 region)	Genus	*Fusobacterium*	32	China	2021	[26]
ESCC	Neoadjuvant camrelizumab and chemotherapy	Camrelizumab plus carboplatin and paclitaxel before surgery	RECIST 1.1	16S rDNA (V3–V4 region)	Phylum	Proteobacteria, Thermi	44	China	2022	[67]
ESCC	Neoadjuvant camrelizumab and chemotherapy	Camrelizumab plus carboplatin and paclitaxel before surgery	RECIST 1.1	16S rDNA (V3–V4 region)	Class	Deinococci	44	China	2022	[67]
ESCC	Neoadjuvant camrelizumab and chemotherapy	Camrelizumab plus carboplatin and paclitaxel before surgery	RECIST 1.1	16S rDNA (V3–V4 region)	Order	Aeromonadales, Pseudomonadales	44	China	2022	[67]
ESCC	Neoadjuvant camrelizumab and chemotherapy	Camrelizumab plus carboplatin and paclitaxel before surgery	RECIST 1.1	16S rDNA (V3–V4 region)	Order	Pseudomonadales	44	China	2022	[67]
ESCC	Neoadjuvant camrelizumab and chemotherapy	Camrelizumab plus carboplatin and paclitaxel before surgery	RECIST 1.1	16S rDNA (V3–V4 region)	Family	Moraxellaceae, Rhodocyclales	44	China	2022	[67]
ESCC	Neoadjuvant camrelizumab and chemotherapy	Camrelizumab plus carboplatin and paclitaxel before surgery	RECIST 1.1	16S rDNA (V3–V4 region)	Family	mitochondria	44	China	2022	[67]
ESCC	Neoadjuvant camrelizumab and chemotherapy	Camrelizumab plus carboplatin and paclitaxel before surgery	RECIST 1.1	16S rDNA (V3–V4 region)	Genus	*Dialister*, *Rhodocyclaceae*, and *Acinetobacter*	44	China	2022	[67]
RCC	TKI (68%)	Nivolumab	RECIST 1.1	Metagenomics	Species	*Erysipelotrichaceae bacterium*_2_2_44A, *Clostridium hathewayi*, and *Clostridium clostridioforme*	58	France	2020	[55]

*HCC* hepatocellular carcinoma, *NSCLC* non-small-cell lung cancer, *GI* gastrointestinal, *ESCC* esophageal squamous cell carcinoma, *RCC* renal cell carcinoma, *ICIs* immune checkpoint inhibitors, *RECIST* Response Evaluation Criteria in Solid Tumors, *PD-1* programmed death 1, *PD-L1* programmed death ligand 1

The common and individual immunotherapy biomarkers can be found among different cancer types (Fig. 2A). For common biomarkers, *Akkermansia muciniphila* had the potential to serve as a common biomarker for responders with liver cancer, lung cancer, or renal cell carcinoma (RCC) [3, 47, 55, 58], and *Faecalibacterium* was enriched in responsive patients with liver cancer, melanoma, or lung cancer [25, 29, 39, 41, 50, 54, 57, 62]. Regarding individual biomarkers, liver cancer patients who responded to immunotherapy exhibited a higher abundance of *Lachnospiraceae bacterium*, *Alistipes* sp. *Marseille*, and *Ruminococcaceae* spp. (at the species level), which were associated with longer PFS and overall survival (OS). Conversely, non-responders showed enrichment of *Veillonellaceae* (at the family level), which is linked to worse PFS and OS [46, 47]. At the species level, responders with lung cancer exhibited increased levels of *Alistipes putredinis*, *Bifidobacterium longum*, *Bacteroides vulgatus*, *Prevotella copri*, and *Parabacteroides distasonis*, while non-responders with reduced PFS demonstrated a decrease in *Ruminococcus unclassified* [48, 49]. At the genus level, *Phascolarctobacterium* and *Ruminococcus* were associated with improved prognosis in lung cancers, while the higher relative abundance of *Dialister* was linked to shorter PFS [61, 62]. For patients with metastatic melanoma, it was observed that *Bifidobacterium longum*, *Bifidobacterium adolescentis*, *Collinsella aerofaciens*, and *Enterococcus faecium* (at the species level) were more abundant in responders as compared to non-responders [65].

Furthermore, Mao and colleagues investigated the correlation between gut microbiota and clinical response to PD-1 inhibitors in patients with hepatobiliary cancers, including HCC and biliary tract cancer (BTC). Their findings suggest that Firmicutes phylum bacteria are more likely associated with a positive immunotherapy response in HCC patients, while Bacteroidetes phylum bacteria are enriched in BTC patients who respond favorably to immunotherapy [46]. In general, the bacterial taxa associated with immunotherapy responses across various tumors do not completely overlap, especially at a lower taxonomic level.

The gut microbiome biomarkers can vary when the kinds of immunotherapy changes. Even the variance in medication dosage can affect the identification of certain microbiome biomarkers (Tables 1, 2). In a prospective study, though *Bacteroides caccae* was enriched in responders regardless of the type of ICI therapy, specific strains were found to be associated with different therapies: *Faecalibacterium prausnitzii*, *Bacteroides thetaiotamicron*, and *Holdemania filiformis* were enriched in Ipilimumab plus Nivolumab responders while *Dorea formicogenerans* increased in pembrolizumab monotherapy responders [39].

The immunotherapy agents may not be the first-line anticancer drugs in clinical scenarios. It is common for cancer patients to receive pre-immunotherapy treatments, including anti-cancer treatment (such as chemotherapies and target therapies) and non-anti-cancer treatment (such as antibiotics treatment for infection). Different studies may involve patients with different pre-immunotherapy treatments [46, 47]. These pre-treatments may have altered the baseline gut microbiome composition of cancer patients, leading to variations in study outcomes. For example, when applied as first-line treatment, tyrosine kinase inhibitor (TKI), one of the most common therapies prior to immunotherapy, has been proven to shift the gut microbiome causing higher enrichment of immunostimulatory *Alistipes senegalensis* and *Akkermansia muciniphila*, both of which are over-present in responders and potentially ameliorate the efficacy of immunotherapy in RCC [55]. Therefore, the pre-treatment of sorafenib can alter the baseline gut microbiome of patients in a different direction compared with no pre-treatment, which may impact the identification of biomarkers for immunotherapy [69]. However, stratified analysis according to specific baseline therapy is difficult, which brings new challenges to the clinical application of immunotherapy biomarkers.

Different sequencing and analyzing methods, as well as diverse reference databases, may also contribute to inconsistent results [70]. Taking sequencing methods as an example, 16-Seq ribosomal RNA gene sequencing (16S rRNA-seq) and metagenomics are the two most commonly used techniques for profiling gut microbiome composition. These two methods can provide microbiome information at different taxonomic levels: 16S rRNA-seq typically identifies up to the genus level, while metagenomic sequencing has the potential to identify species.

The situation is particularly perplexing as bacteria species within the same genus may exhibit opposite effects. For instance, *Bacteroides zoogleoformans* was associated with improved responses to immunotherapy, while *Bacteroides ovatus*, *Bacteroides dorei*, and *Bacteroides massiliensis* were related to worse PFS [46, 50] (more examples in Tables 1, 2). These results suggest that we should interpret the results of immunotherapy biomarkers with caution, as differences in bacterial species, even within the same genus, can lead to opposite conclusions, so caution should be exercised with cross-genus or cross-species generalization of any microbiome biomarker.

It is well known that gut microbiome is subject to numerous influences, including some clinical factors that are often neglected in the experiments. In immunotherapy, the size, number, and stage of tumors were found positively correlated with the responder-related gut microbiome in liver cancer, whereas patients with poor liver function and elevated levels of bile acid and bilirubin tend to exhibit a higher prevalence of non-responder-related gut microbiome [46]. Other confounding factors, such as dietary, seasons, and geographical locations, may also influence the gut microbiome. For instance, Fang et al. compared the gut microbiome of Chinese and French NSCLC cohorts and observed that the strains of *Akkermansia muciniphila*, which were abundant in responders, differed between the two groups (with those from France belonging to MGS.igc0118 and those from China belonging to MGS.igc0776) [58].

In addition to the abundance of specific gut microbiota, the relative abundance ratio among different bacteria is also a potential predictor of immunotherapy response. A proper Firmicutes/Bacteroidetes ratio (phylum level) (generally 0.5–1.5), as well as a higher *Prevotella*/*Bacteroides* ratio (genus level), was found more frequently in the responders with HCC [53]. Moreover, most of the studies focus on the fecal microbiome due to its easy access and convenient detection. However, the microbiome in other parts of the GI is also important for immunotherapy. For example, *Helicobacter pylori* is an important pathogen mainly cloned in the stomach, and a recent study found that *H. pylori* seronegative patients survive longer than seropositive patients (survival median: 6.7 months compared with 15.4 months) in NSCLC patients treated with PD-1 inhibitors [71]. Additionally, it should be noted that certain local tumor microbes have been found to correlate with the response to immunotherapy and may serve as prognostic indicators for immunotherapy, although it is not the primary focus of this review. For instance, a high diversity of local NSCLC microbiota was associated with improved prognosis, while Gammaproteobacteria in local tumor tissues were linked to low PD-L1 expression and unfavorable results from immunotherapy [72].

### The functional components of the gut microbiome

The microbiome exerts its functions through gene expression, encompassing transcription and translation processes. The pathways and products involved during these processes may serve as potential biomarkers for immunotherapy. Peters et al. incorporated meta-transcriptomics into their study on the association between gut microbiome and immunotherapy responses in melanoma patients for the first time. The pathways exhibiting consistent positive associations between metagenomic and meta-transcriptomic expression were identified and classified into protective pathways, such as biosynthesis of l-isoleucine and petroselinate, associated with longer PFS, and risk-associated pathways linked to shorter PFS, including guanosine nucleotide biosynthesis, l-rhamnose degradation, and B vitamin biosynthesis [50]. Notably, a positive correlation was observed between risk-associated pathways and unfavorable bacterial species, and a negative correlation was found between risk-associated pathways and protective bacteria species. Nevertheless, no significant association was shown between protective pathways and protective species [50], suggesting that these protective pathways may serve as independent predictive factors regardless of taxonomic compositions. The transcriptomic differences between immunotherapy responders and non-responders were also observed in a United States NSCLC cohort. Specifically, thirty genes were significantly upregulated in responders while ten genes were upregulated in non-responders [62]. Further analysis revealed that carbon fixation pathways were particularly abundant among responders, whereas phosphotransferase systems were more prevalent among non-responders [62].

The metabolic pathways and products of the gut microbiome can also function as biomarkers for immunotherapy. In a study utilizing PD-1 inhibitors for NSCLC, Song et al. conducted an analysis of the functional group protein family and gut microbiome metabolism in patients with different PFS (≥ 6 months or < 6 months). The metabolic potential of methanol and methane, as well as 390 (KO), 264 (COG), and 859 (CAZy) functional group abundances, were found to have significant differences between the two groups [51]. In patients with liver cancer, the gut microbiome of immunotherapy responders was more likely to be associated with energy metabolism based on functional annotation, while amino acid metabolism was linked to non-responders [46]. SCFAs are important fermentation products of non-digestible carbohydrates by gut microbiota, exerting significant impacts on human health [73]. In a study involving eleven patients treated with Nivolumab (PD-1 inhibitors), early NSCLC progression was significantly associated with 2-pentanone and tridecane, while butyrate, propionate, lysine, and nicotinic acid were more likely to be related to favorable outcomes [74]. Bile acids, another type of gut microbiome metabolic product, have also been found to be associated with the efficacy of immunotherapy. Responders in HCC patients were observed to have significantly higher levels of secondary bile acids (such as UDCA and UCA), which are synthesized from primary bile acids by gut bacteria [37].

### Summary

In short, the characteristics of the gut microbiome at baseline are promising biomarkers for predicting the efficacy of immunotherapy. However, the results of various studies are not always inconsistent, and even within the same type of cancer, a uniform or universal conclusion has not been drawn [48, 49, 61, 75, 76]. The inconsistent results may be attributed to the variation of (i) cancer types, (ii) analysis methods, (iii) sample size, (iv) types of immunotherapies/drugs, (v) pre-treatments, (vi) clinical factors of patients, and (vii) other confounding factors (Fig. 3). To get conclusive outcomes and put them into clinical applications, more dedicated designing of trials, larger scales of participants, and more up-to-date inter-disciplinary methods are in urgent need. Table 3 summarizes some of the clinical trials aimed at identifying appropriate gut microbiome-derived biomarkers.

**Table 3:** Clinical trials of gut microbiome as biomarker of immunotherapy

Type	Conditions	Interventions	Phases	Enrollment	Funder type	Start date	Completion date	Locations	Study status	NCT number
Response	Gastric cancer, stomach neoplasm	JS001 + chemotherapy (XELOX or SOX)	Phase2	110	Other	2021/3/12	2024/12/30	China	Recruiting	NCT04744649
Response	ESCC	Sintilimab + chemotherapy	–	30	Other	2021/12/1	2023/6/1	China	Recruiting	NCT05199649
Response	Colorectal adenocarcinoma	Fruquintinib + PD-1 inhibitors; fruquintinib + PD-1 inhibitors + radiotherapy	–	100	Other	2022/1/1	2023/9/30	China	Recruiting	NCT05635149
Response	CRC	CAP + pembrolizumab + bevacizumab	–	50	Other	2018/4/13	2022/6/30	US	Completed	NCT04054908
Response	Upper GI cancer	Immunotherapy	–	40	Other	2021/10/1	2023/12/1	China	Recruiting	NCT05065515
Response	Metastatic carcinoma, CRC	Chemotherapy/immunotherapy	–	21	Other	2016/10/20	2022/4/13	US	Terminated	NCT02960282
Response	NSCLC	PD-1/PD-L1 inhibitors	–	50	Other	2021/8/12	2022/12/30	China	Recruiting	NCT04682327
Response	Metastatic NSCLC	ICI; ICI + chemotherapy	–	24	Other	2021/5/3	2025/11/1	France	Active not recruiting	NCT04804137
Response	NSCLC, CRC, TNBC, pancreas cancer	Immunotherapy; chemotherapy	–	5000	Industry	2022/7/1	2025/12/31	US	Recruiting	NCT04638751
Response	NSCLC, malignant melanoma, RCC, TNBC	ICI	–	800	Industry	2021/11/22	2028/9/14	US	Recruiting	NCT05037825
Response	Carcinoma	Pembrolizumab	–	100	Industry	2019/6/28	2022/12/31	US	Unknown	NCT04291755
Response	Melanoma	ICI	–	450	Other_gov	2018/4/4	2023/5/2	UK	Unknown	NCT03643289
Response	Urothelial carcinoma	Atezolizumab; pembrolizumab	–	40	Other	2020/12/2	2023/6/1	France	Recruiting	NCT04566029
Response	Advanced cancer	ICI	–	150	Other	2016/11/29	2024/9/1	US	Recruiting	NCT04204434
Response	Diffuse large B cell lymphoma	Chemo-immunotherapy	–	50	Other	2019/4/2	2023/12/20	Italy	Active not recruiting	NCT03797170
Response	Gynecologic cancer	Immunotherapy	–	30	Industry	2021/6/29	2023/6/1	US	Recruiting	NCT04957511
Response	Melanoma	Pembrolizumab; lenvatinib	Phase2	44	Other	2023/5/1	2030/10/1	Australia	Not yet recruiting	NCT05545969
Response	Solid carcinoma	Immunotherapy	–	60	Other	2018/6/4	2023/4/30	Korea	Unknown	NCT04264975
Response and irAEs	Melanoma, renal cancer, lung cancer	Nivolumab; pembrolizumab; ipilimumab; durvalumab; tremelimumab; atezolizumab; bevacizumab	–	1800	Other	2020/7/8	2025/7/8	UK	Recruiting	NCT04107168
Response and irAEs	Non-squamous NSCLC	Pembrolizumab; pembrolizumab + pemetrexed + carboplatin	–	150	Other	2021/5/18	2024/12/31	US	Recruiting	NCT04954885
Response and irAEs	Advanced solid tumor	ICI	–	60	Other	2018/4/1	2024/12/1	Canada	Recruiting	NCT04579978
Response and irAEs	Lung cancer	ICI	–	44	Other	2018/8/30	2022/5/9	US	Completed	NCT03688347
irAEs	Melanoma	Ipilimumab; nivolumab; pembrolizumab	–	123	Other	2013/8/1	2019/4/1	Netherlands	Completed	NCT02600143
irAEs	NSCLC	ICI	–	150	Other	2021/1/21	2023/3/3	US	Recruiting	NCT04913311

*US* United States, *UK* United Kingdom, *GI* gastrointestinal, *ESCC* esophageal squamous cell carcinoma, *NSCLC* non-small cell lung cancer, *TNBC* triple negative breast cancer, *CRC* colorectal cancer, *CAP* capecitabine, *RCC* renal cell carcinoma, *ICI* immune checkpoint inhibitor

## Prediction of irAEs by gut microbiome

The activation of immune response by immunotherapy may result in the loss of control over the immune system, leading to irAEs [23, 77]. Prediction of the irAEs, especially the severe events, is crucial for preemptive prevention and optimal application of immunotherapy. The incidence of irAEs was found to be comparable among NSCLC patients with different levels of PD-L1 expression (≥ 1% or < 1%) in a phase three clinical trial [18], indicating that molecular markers may not be reliable predictors for irAEs. Conversely, the microbiota appears to have a more significant role in predicting irAEs.

The gut microbiome can be both risk factors and protective factors for irAEs. Checkpoint inhibitor colitis (CIC) is the most frequently reported irAE. *Faecalibacterium prausnitzii* acts as a risk factor in CIC while *Bacteroides fragilis* is deemed to be a protective factor due to its anti-inflammatory role in the GI tract [23, 25, 45]. The enrichment of Bacteroidetes, which is a proposed immune regulator and can reduce inflammation by promoting Treg differentiation, can also be the marker of resistance to the development of CIC [25, 45]. In liver cancer, the reduction of diversity and relative abundance in the gut microbiome was associated with severe immunotherapy-related colitis [46], which implied that not only can the gut microbiome predict irAEs, but also their severity. In metastatic melanoma patients receiving Ipilimumab, a higher abundance of *Faecalibacterium* and other Firmicutes was associated with better response (longer PFS and OS) and more colitis with a low proportion of Treg in peripheral blood [25], indicating that specific bacteria may predict both the efficacy and irAEs of immunotherapy. As for the functional components of gut microbiome, two pathways (polyamine transport system and biosynthesis of B vitamins) were found related to colitis-free patients with melanoma [45].

In addition to CIC, the gut microbiome can also predict other irAEs, such as diarrhea and skin toxicity [78]. *Prevotellamassilia timonensis*, which are potential biomarkers to predict the severity of immunotherapy-related colitis in liver caner, were also found to be enriched in cases with severe diarrhea [46]. Immunotherapy-related skin toxicity in advanced NSCLC patients was significantly linked to a decreased diversity of gut microbiome [61]. In a prospective cohort study (NCT03688347), it has been found that the overall irAEs rather than one type of irAEs can be predicted by some bacteria, such as *Bifidobacterium* and *Desulfovibrio* [64]. More clinical trials involving gut microbiome as a biomarker of immunotherapy and irAEs were summarized in Table 3.

In recent years, neoadjuvant immunotherapy has emerged as a rapidly developing treatment option for cancer patients [79]. Xu et al. found that taxonomic features of the gut microbiome can predict the pathological response and severe adverse events (≥ 3 grade) in esophageal squamous cell carcinoma (ESCC) patients who were undergoing neoadjuvant camrelizumab and chemotherapy [67], which further expands the potential applications for gut microbiome biomarkers.

## Manipulation of the gut microbiota to enhance immunotherapy

Compared with other response markers for immunotherapy, the gut microbiome not only serves as a fascinating biomarker but also as an intervention target [80]. Manipulation of the gut microbiome can increase the proportion of responders, enhance therapeutic benefits, and mitigate severe adverse events in immunotherapy. The manipulation methods of the gut microbiota could be divided into overall manipulation (such as FMT or antibiotics usage) and specific manipulation (such as probiotics/prebiotics supplement or selective antibiotics usage) (Fig. 2B). Herein, we concentrate on the latest advancements, obstacles, and prospects in manipulating the gut microbiome to augment immunotherapy.

### Probiotics supplementation

Oral administration of specific members of gut microbiota (probiotics) is a convenient and acceptable method for manipulating the gut microbiota. The classical probiotics mainly belong to *Lactobacillus* or *Bifidobacterium* [81]. It has been proved in mouse models that oral administration of *Bifidobacterium* spp. can enhance the efficacy of PD-L1 inhibitors and almost eliminate tumor outgrowth, which was mediated by the activation of DCs and subsequent enhancement of tumor-specific CD8^+^ T cells [82]. Interestingly, the administration of *Bifidobacterium* alone is sufficient to achieve comparable results in tumor control as PD-L1 inhibitors alone [82], suggesting a synergistic effect of microbiome and immunotherapy. *Lactobacillus rhamnosus* is another widely-used probiotic that can rescue the poor efficacy of ICIs treatment caused by prior antibiotic intake. The *L. rhamnosus* not only synergized with ICI therapy and recovered the diversity and composition of gut microbiome but also increased the enrichment of favorable bacteria (such as *Bifidobacterium pseudolongum* and *Bacteroides*) [83]. In a multicenter retrospective study, it was also demonstrated that the use of traditional probiotics in NSCLC patients treated with anti-PD-1 monotherapy was associated with a favorable prognosis [84].

With the development of microorganism culturing and gene sequencing methods, an increasing number of microorganisms have been identified as potentially beneficial organisms for humans. These microorganisms are referred to as next-generation probiotics (NGPs), such as some bacteria species from *Akkermansia*, *Bacteroides*, and *Faecalibacterium* [81]. These NGPs are also potential manipulated factors for better immunotherapy efficacy. *Akkermansia muciniphila*, an NGP and health-promoting mucin degrader, can retrieve the efficacy of PD-1 inhibitors by recruiting CCR9^+^CXCR3^+^CD4^+^ T cells in mice transplanted with feces of non-responders [3, 85]. Another NGP, *B. fragilis*, was also found to be effective in restoring the impaired antitumor effects of CTLA-4 blockade in antibiotic-treated mice [33]. *Clostridium butyricum* is a probiotic bacterium that can increase the abundance of other probiotics and promote the expansion of IL-17A-producing cells (including γδT cells and CD4 cells) [86]. In a clinical trial (NCT03829111), RCC patients who received a combination therapy of ICIs and CBN588, a live bacterial product containing *Clostridium butyricum*, demonstrated significantly longer PFS compared to those who received ICIs without CBM588 (12.7 versus 2.5 months) [87]. In addition to a single strain of bacteria, a collection of bacteria strains may also cooperate to enhance the antitumor immunity and therapeutic effects of immunotherapy. A combination of eleven bacterial strains was found to act together and induce interferon-γ-producing CD8^+^ T cells without causing inflammation, thereby enhancing ICIs efficacy in mice models [88].

Collectively, in cases where the mechanisms of the gut microbiome’s influence on immunotherapy are clear, it is highly desirable to improve immunotherapy outcomes by supplementing with probiotics, as the addition of probiotics can more specifically alter the structure of the gut microbiome. However, even traditionally used probiotics should be treated with caution, as the inappropriate use of probiotics may compromise the efficacy of immunotherapy and even promote tumorigenesis. In a cohort of 158 melanoma patients treated with ICIs, no statistically significant differences were observed between those who received probiotics and those who did not. Surprisingly, patients who did not take probiotics had better outcomes (probiotics versus non-probiotics: PFS 17 versus 23 months; response rate 59% versus 68%) [89]. In further preclinical models, it has been observed in different models that mice receiving probiotics showed remarkably larger tumors and impaired antitumor response to immunotherapy [89]. In line with these findings, it is observed in a human cohort study that patients who consumed an adequate amount of fiber without using probiotics exhibited the most significant improvement in melanoma immunotherapy compared to other groups (PFS not reached versus 13 months; response rate 82% versus 59%) [89].

### Prebiotics and dietary fibers supplementation

Particular substances, such as dietary fibers and prebiotics, can improve the efficacy of immunotherapy by altering the gut microbiome. Prebiotics is “a substrate that is selectively utilized by host microorganisms conferring a health benefit” [90]. Diosgenin, which is derived from yam, has prebiotic effects and can promote the growth of lactic acid bacteria (such as *Lactobacillus murinus* and *Lactobacillus reuteri*) in GI tracts [91]. In melanoma C57BL/6 mouse models, diosgenin administration enhanced the efficacy of PD-1 antibody by modulating intestinal microbiota and stimulating T-cell responses [92]. Ginseng polysaccharides (GPs), the most essential components of traditional Chinese medicine *Panax ginseng*, have potential prebiotic properties. A recent study found that GPs improved the effect of αPD-1 monoclonal antibody (mAb) by modulating gut microbiome metabolites such as valeric acid and l-kynurenine [49].

Dietary fibers, mainly found in plants, are indigestible polysaccharides for humans. However, gut bacteria can break them down through fermentation and produce many useful products such as SCFA [93]. In an observational study, the researchers discovered that patients with melanoma who reported sufficient fiber consumption responded to ICIs better than those who reported a diet with insufficient-fiber [89]. Delayed tumor outgrowth was also observed in melanoma mouse models supplied with sufficient fibers, while this effect did not arise in germ-free mice, indicating that this effect of dietary fiber depended on gut microbiome [89]. Pectin, a type of soluble fiber, can enhance the efficacy of PD-1 inhibitors by increasing T cell infiltration. Further study found that the alteration of gut microbiome and butyrate might play pivotal roles in mediating this ameliorative effect [94].

### FMT

FMT, which transfers gut microbiome from one person to another, is a valuable treatment for recurrent *Clostridium difficile* infection and has shown an effective role in reconstructing and improving gut microbiome and immune system [95, 96]. The advantage of FMT is that the gut microbiome is intervened as a whole, which can be used even when the mechanism is unclear. Lots of preclinical research based on animal models has proved the effectiveness of FMT in increasing the sensibility of immunotherapy and turning the cancer models from non-responders to responders [29, 65]. Routy et al. found that antibiotic-induced dysbiosis may reduce the efficacy of ICIs in mice epithelial tumors, while FMT can recover it [3]. FMT has also been proven to be effective in addressing irAEs, as evidenced by the successful treatment of two patients with refractory ICI-associated colitis who experienced complete resolution of clinical symptoms following FMT intervention [97].

Despite the benefits of FMT, mice models receiving gut microbiome from humans have revealed a discrepancy in immunotherapy responses between FMT donors and recipients. The response mismatching group (1/3) showed significantly different gut microbiota compositions between the mice recipients and human donors, and the binary Bray–Curtis dissimilarity index of mismatching donor/recipient pair was high (0.7) compared with the matching groups (0.5 to 0.6) [65]. A possible explanation is that the FMT cannot always guarantee the accurate transfer of gut microbiome from donors to recipients, so the gut microbiome may drift to a large degree in recipients compared with donors. Different responders may have different beneficial microorganisms, and some favorable microorganisms may be challenging to be transferred from donors to recipients due to some reasons such as belonging to obligate anaerobes that may die during the FMT process. Hence, it is crucial to develop new methods or procedures for FMT that can maximize the reconstitution of the gut microbiome in recipients.

The response mismatches between donors and recipients were also found in human trials. Recently, two clinical trials have evaluated the safety and efficacy of transferring fecal microbiota from anti-PD-1 responders to non-responders [96, 98]. Davar et al. found that among 15 melanoma patients who were resistant to PD-1 inhibitors, six of them exhibited clinical benefits after receiving FMT [98]. Among the six patients who got benefits, three showed objective responses (ORs), while the remaining three showed stable disease (SD) for more than 12 months. Intriguingly, though there are seven donors in total, all three recipients who turned into responders received gut microbiome from an identical donor [98]. In another phase one clinical trial, the security and feasibility of FMT were demonstrated in patients with metastatic melanoma [96]. Two patients who had received PD-1 inhibitors and achieved complete response (CR) for at least 1 year were selected as donors in this trial. Their gut microbiomes were separately transplanted to five recipients who did not respond to anti-PD-1 therapy. Three of the recipients achieved responses after FTM with only mild adverse events, and all three responsive recipients received gut microbiome from the same donor (donor 1). The gene sets analysis demonstrated that donor 1-group recipients upregulated some immune-related gene sets (such as antigen-presenting cell (APC) activity, innate immunity, and IL-12) while donor 2-group recipients did not [96]. Gopalakrishnan et al. analyzed the fecal microbiome of melanoma patients undergoing anti-PD-1 immunotherapy (n = 43, responders:non-responder = 30:13). The patients were separated into two distinct communities by unsupervised hierarchical clustering of crOTU abundances without the input of response data. The first community is composed entirely of responders, while the second community consists of both responders and non-responders, suggesting that some responders may share similar gut microbiome features with non-responders [29]. These results suggest that different mechanisms may underlie patient response, either dependent on the gut microbiota or primarily driven by other factors (such as the expression of PD-L1). Thus, one possible hypothesis is that only the responders relying on unique gut microbiome features different from non-responders would be suitable and effective candidates for FMT donors.

In conclusion, the immunotherapy response in recipients after FMT sometimes, but not always, paralleled the clinical response of the donors. Though limited by the sample size, these results still clued that the donors (responders) of FMT should be selected carefully. Therefore, we proposed that classification among the responders according to the gut microbiome, as well as the screening criteria of FMT donors, should be further explored in the future.

### Antibiotics usage

Antibiotics are frequently administered prior to or during cancer immunotherapy, which can significantly alter the gut microbiome and lead to dysbiosis characterized by reduced bacterial diversity and altered composition of the gut microbiota. Given the significant impact of gut microbiota on cancer immunotherapy, exploring the correlation between antibiotics and immunotherapy efficacy is particularly intriguing. Most of the research showed that antibiotic administration is harmful to immunotherapy in different kinds of cancers, such as melanoma, lung cancers, and renal cancers [99–103]. However, divergent perspectives were proposed in liver cancer recently [104, 105]. The dual function of antibiotics and the unique characteristics of liver cancer in immunotherapy will be discussed in this section.

#### The harmful effect of antibiotics on immunotherapy

The usage of antibiotics has been reported to be associated with various cancer risks and metastasis [106]. The impaired efficacy and worse clinical outcomes of immunotherapy caused by antibiotic use were found in both animal models and patients. For example, a study involving 249 patients with NSCLC, RCC, or urothelial carcinoma found that those who took antibiotics from 2 months before to 1 month after the first dose of immunotherapy had significantly shorter PFS (3.5 versus 4.1 months; p = 0.017) and OS (11.5 versus 20.6 months; p < 0.001) compared to those who did not take antibiotics [3]. In a study of advanced RCC patients treated with Nivolumab, the use of antibiotics resulted in a reduction of response rate from 28 to 9%, as well as decreased PFS and OS. Furthermore, the over-represented species of gut microbiota in the antibiotics-usage group changed to *Clostridium hathewayi* and *Erysipelotrichaceae bacterium*_2_2_44A, both of which were enriched among non-responders in the cohort without antibiotic use [55].

The duration between antibiotic administration and immunotherapy has an impact on the interference of antibiotics with immunotherapy. A cohort study (n = 196) revealed that prior use of antibiotics in NSCLC, melanoma, or other cancers was associated with poorer outcomes of immunotherapy, while concurrent use did not show such association. This highlights the importance of considering the timing effect when using antibiotics in conjunction with immunotherapy [99]. To confirm the timing effect of antibiotic use, Derosa et al. compared the impact of antibiotic usage within 30 days or 60 days of starting ICIs in RCC or NSCLC patients. The study revealed that while adverse effects caused by antibiotics persisted in patients receiving antibiotics 60 days before ICIs initiation, the extent of their impact was less severe than those who took antibiotics within 30 days. These differences may be attributed to the partial restoration process of gut microbiota [22]. A similar phase 1 clinical trial was conducted in patients with advanced cancers, including RCC, NSCLC, melanoma, sarcoma, GI stromal tumors. Patients who received antibiotics within 30 days before initiation of ICIs showed significantly worse OS, while there was no difference in OS for those who received antibiotics during ICI use or 30–60 days before ICI [100]. In conclusion, the effect of antibiotic on immunotherapy are limited in a specific period before initialing immunotherapy (neither earlier nor later than this time frame will be effective). Possible explanations are that it takes time for gut microbiota to modulate the immune system after antibiotic administration and that the altered gut microbiome can gradually recover over time.

Different types and dosages of antibiotics may have varying impacts on the effectiveness of immunotherapy. Ahmed et al. found that broad-spectrum antibiotics were associated with a lower response rate and longer response time, whereas narrow-spectrum antibiotics did not affect the response rate [107]. In a retrospective cohort study of 2737 cancer patients receiving ICIs, exposure to fluoroquinolones was found to be associated with OS, and a dose–response relationship was observed, while no association was found between exposure to penicillin or cephalosporin and OS [102]. In patients diagnosed with non-Hodgkin lymphoma (NHL), exposure to different antibiotics, including “P-I-M” (piperacillin/tazobactam, imipenem/cilastatin, and meropenem) or cefepime within 4 weeks before chimeric antigen receptor (CAR) T cell treatment, was found to have varying effects on OS [66]. Specifically, individuals exposed to P-I-M had a higher hazard ratio (HR = 3.32) than those who were not exposed while cefepime exposure resulted in a lower HR (0.69) when compared to the unexposed group. Moreover, P-I-M exposure was linked to worse OS (HR = 2.19) compared to exposure to non-P-I-M antibiotics though shorter PFS did not reach a statistical significance [66]. These results underlined the clinical significance of selecting appropriate antibiotics for prospective recipients of immunotherapy. Antibiotic exposure was associated with not only poor survival but also immunotherapy toxicities.

In a retrospective B cell malignancies cohort (n = 228), antibiotics used within the 4 weeks before CAR T cell infusion was significantly correlated with increased incidence of neurotoxicity [immune effector cell-associated neurotoxicity syndrome (ICANS)] and worse survival outcomes characterized by shorter OS (HR = 1.71) in CD19-targeted CAR T cell therapy [66]. However, in further subgroup analysis, the association between antibiotics and ICANS was observed in NHL but not in ALL, indicating a potential cancer type heterogeneity [66].

#### The beneficial effect of antibiotics on immunotherapy

Despite the extensive harmful effect of antibiotics on immunotherapy in various types of cancers, things seem different in liver cancers. In an international cohort containing 450 HCC patients in 12 centers from different continents, antibiotic exposure during the early immunotherapy period (EIOP)—defined as 30 days before or after initiation ICIs—was found to correlate with improved benefit from ICIs (better PFS, as well as similar OS, response rate, and disease control rates) [104]. Moreover, diverse outcomes were observed among different immunotherapies and antibiotics. The correlation between antibiotic exposure and higher disease control rates as well as longer PFS was found in patients restricted to PD-1/PD-L1 monotherapy [104]. A sub-group analysis was conducted on different classes of antibiotics, including beta-lactams, quinolones, other single-agent antibiotics, and antibiotic combinations. Only patients receiving quinolones were reported to have a significantly prolonged PFS [104]. Furthermore, early exposure to antibiotics remained a significant independent predictor of PFS in multivariable models that accounted for the severity of chronic liver disease, performance status, and HCC stage [104]. This finding provides genuine pathophysiological evidence rather than just an associative link between antibiotic use and improved disease control during ICI therapy [104]. Though the OS did not improve, there may still be potential benefits of antibiotics on OS which may be masked by death from liver decompensation and worsening chronic liver disease [104]. The potential beneficial effect of antibiotics in liver cancer immunotherapy may come from the unique immunity of liver. Liver is recognized to be a special organ with immune privilege (also termed liver tolerance) [108, 109]. For example, the APCs in liver show immunosuppressive action [97, 110]. Besides, prolonged exposure to antigens induced expression of immunosuppressive checkpoint molecules and T cell exhaustion in liver [111–113]. These immune tolerance processes may be suppressed by the bacteria and antigen clearance effect of antibiotics, which may play a synergistic role with immunotherapy in anti-tumor therapy. Therefore, manipulating gut microbiome via antibiotics may serve as a novel approach to enhance immunotherapy for liver cancer. However, there are still many challenges before its clinical application. First, more evidence, especially experimental ones, is needed to further verify the effect of antibiotics on liver cancer, as most of the current evidence is observational. Second, as mentioned earlier, different types, dosage forms, and duration of antibiotics will affect the therapeutic effect of antibiotics. Determining when, where, and how these possible effects could be utilized to enhance immunotherapy efficacy is possibly an important concern in future studies. Third, adverse events and antibiotic resistance from antibiotic treatment could not be neglected, especially with the long-term administration of broad-spectrum antibiotics and antibiotic cocktails which may eliminate almost all of the commensal microbiota [114]. To minimize toxicity and reduce antibiotic resistance, selective antibiotics targeting harmful bacteria or metabolism may be a better alternative. Fourth, many patients with liver cancer have liver dysfunction or need to use antibiotics due to infection. In clinical practice, attention should be paid to balance the relationship between antibiotics used to increase the efficacy of immunotherapy and antibiotics used for other reasons. That is, selective antibiotics of less toxicity and less risk of gaining resistance, could be considered to be potential agents in pre-immunotherapy treatment.

In summary, the principle of manipulating gut microbiota to enhance the efficacy of immunotherapy can be summarized as follows: when the functional microorganisms or mechanisms are clearly defined, increasing favorable microbes (i.e., through probiotics/prebiotics supplementation) or reducing harmful microbes (i.e., through selective antibiotic usage) can be chosen. However, when the mechanisms are unclear, considering the integrality of the gut microbiome (i.e., through FMT or broad-spectrum antibiotic usage) should be taken into account (Fig. 2B). Remarkably, although antibiotics have harmful effects on immunotherapy for most cancer types, their application in liver cancer prevention and immunotherapy enhancement has shown promising results [104, 114]. Table 4 summarizes clinical trials regarding gut microbiome manipulation to enhance the efficacy of immunotherapy. In conclusion, despite ongoing challenges, there is considerable potential for using gut microbiota in clinical practice.

**Table 4:** Clinical trials of manipulating the gut microbiota to enhance immunotherapy

Type	Conditions	Interventions	Phases	Enrollment	Funder type	Start date	Completion date	Locations	Study status	NCT number
Probiotics	RCC	CBM 588 + ipilimumab + nivolumab	Phase1	30	Other	2019/5/14	2023/6/11	US	Active not recruiting	NCT03829111
Probiotics	Advanced HCC	Bifidobacterium bifidum + carrilizumab + apatinib mesylate	Phase1|phase2	30	Other	2021/11/1	2024/10/31	China	Recruiting	NCT05620004
Probiotics	NSCLC	*Lactobacillus bifidobacterium* V9(Kex02) + carilizumab + platinum	–	46	Other	2021/10/19	2023/12/30	China	Recruiting	NCT05094167
Probiotics	Melanoma	Pembrolizumab + EDP1503	Phase2	8	Other	2018/10/2	2023/11/2	US	Suspended	NCT03595683
Probiotics	Liver cancer	M9 + PD-1 inhibitors	–	46	Other	2021/11/19	2023/12/30	China	Recruiting	NCT05032014
Probiotics	Metastatic melanoma	SER-401 + nivolumab	Phase1	14	Other	2019/1/28	2022/3/4	US	Completed	NCT03817125
Probiotics	All solid tumors	MET-4 + ICI	Phase2|phase3	65	Other	2018/11/30	2025/12/1	Canada	Active not recruiting	NCT03686202
Probiotics	NSCLC, melanoma, RCC	BMC128 + nivolumab	Phase1	12	Industry	2022/5/1	2023/5/1	Israel	Recruiting	NCT05354102
Probiotics	RCC	*Clostridium butyricum* CBM 588 + nivolumab and cabozantinib	Phase1	30	Other	2021/11/1	2023/11/30	US	Recruiting	NCT05122546
FMT	GI system cancer	FMT + PD-1 inhibitors	Phase1	10	Other	2020/1/3	2021/12/1	China	Unknown	NCT04130763
FMT	RCC	FMT + ICI	Phase1|phase2	50	Other	2021/2/18	2024/2/19	Italy	Recruiting	NCT04758507
FMT	Mesothelioma	FMT + keytruda	Early_phase1	1	Other	2018/9/18	2018/12/18	US	Completed	NCT04056026
FMT	Advanced HCC	FMT + atezolizumab + bevacizumab	Phase2	48	Other	2024/1/1	2027/3/1	Germany	Not yet recruiting	NCT05690048
FMT	Cancer	FMT + ICI	–	30	Other	2022/3/14	2025/3/31	Switzerland	Recruiting	NCT05273255
FMT	HCC	FMT + atezolizumab + bevacizumab	Phase2	12	Other	2023/3/1	2026/1/1	Austria	Not yet recruiting	NCT05750030
FMT	RCC	FMT + nivolumab + ipilimumab	Phase1	20	Other	2020/1/23	2028/11/1	Canada	Recruiting	NCT04163289
FMT	Melanoma	FMT + ICI	Phase1|phase2	24	Other	2022/7/1	2025/4/1	Netherlands	Recruiting	NCT05251389
FMT	NSCLC	FMT + PD-1/PDL-1 inhibitors	Phase1	20	Other	2021/9/1	2022/12/30	China	Not yet recruiting	NCT05008861
FMT	Melanoma	FMT + ICI	Phase1	40	Other_gov	2017/11/30	2021/12/30	Israel	Unknown	NCT03353402
FMT	Metastatic lung cancer	FMT + ICI	Phase2	80	Other	2022/9/1	2028/6/30	Israel	Not yet recruiting	NCT05502913
FMT	Lung cancer	FMT + PD1 inhibitors	–	20	Other	2021/4/23	2022/12/31	Spain	Recruiting	NCT04924374
FMT	Melanoma	FMT + pembrolizumab/nivolumab	Phase1	20	Other	2019/3/27	2023/12/1	Canada	Active not recruiting	NCT03772899
FMT	Solid carcinoma	FMT + immunotherapy	–	60	Other	2018/6/4	2023/4/30	Korea	Unknown	NCT04264975
Antibiotics	Pancreatic cancer	Antibiotics + pembrolizumab	Phase4	0	Other	2019/6/25	2020/6/1	US	Withdrawn	NCT03891979
Antibiotics	Pancreatic cancer	Chemotherapy + antibiotics + pembrolizumab	Phase2	25	Other	2022/8/1	2029/4/1	US	Not yet recruiting	NCT05462496
Diet	Head and neck cancer	Prolonged nightly fasting + ICI (nivolumab/pembrolizumab/atezolizumab/avelumab/durvalumab)	–	29	Other	2021/10/20	2023/5/24	US	Active not recruiting	NCT05083416
Diet	Solid tumor	Potato starch + ICIs	Early_phase1	12	Other	2021/6/2	2023/1/24	US	Completed	NCT04552418
Diet	Lung cancer	Fish oil + immunotherapy/chemotherapy/TKI	–	50	Other	2022/11/15	2024/5/15	Brazil	Recruiting	NCT04965129
Diet	Melanoma	Dietary intervention + pembrolizumab/nivolumab	Phase2	42	Other	2020/6/24	2024/2/1	US	Recruiting	NCT04645680
Diet and exercise	Melanoma	Immunotherapy	–	80	NIH	2023/5/15	2025/10/31	US	Recruiting	NCT04866810

*US* United States, *GI* gastrointestinal, *NSCLC* non-small cell lung cancer, *RCC* renal cell carcinoma, *HCC* hepatocellular carcinoma, *ICI* immune checkpoint inhibitor, *FMT* fecal microbiota transplantation, *TKI* tyrosine kinase inhibitor

## Further directions

### Large sample size

Clinical studies with large sample sizes are required to better illustrate the connection between the human gut microbiota and the therapeutic effects of immunotherapy. While general biomarkers or manipulation methods may seem more attractive, more precise subgroup analyses based on large sample sizes are necessary. Large samples not only aid in obtaining consistent results but also form the foundation for detailed analyses. Biomarkers may be only suitable for certain cancer types, immunotherapies, pathological patterns, or drug dosages due to the heterogeneity of cancers and immunotherapy. For instance, the level of PD-L1 expression showed a predictive association with the benefits from Nivolumab in nonsquamous NSCLC, but not in squamous-cell NSCLC [18, 115]. The individual health states of patients (such as the immune states), as well as other confounding factors that may influence the gut microbiome of the host, should also be considered. In addition, the response could be classified into CR, partial response (PR), SD, and progressive disease (PD) based on Response Evaluation Criteria in Solid Tumors (RECIST) with sufficient sample size, rather than being simply dichotomized into responders (CR, PR, and SD) and non-responders (PD). Although most clinical trials investigating the role of the gut microbiome in immunotherapy involve no more than 100 patients (Tables 1, 2), several trials with large sample sizes are currently underway. A large-scale, prospective MITRE trial (NCT04107168) across three types of cancers, including melanoma, renal cancer, and lung cancer, intends to enroll 1800 participants (Table 3) [76]. Additionally, a multicenter, prospective, observational study (trial registration number: UMIN000046428) involving 400 lung cancer patients aims to identify gut microbiome predictive biomarkers of immunotherapy response using artificial intelligence and is scheduled to be completed in 2024 [75].

### Combination immunotherapy

In addition to monotherapy, immunotherapy can be combined with other immunotherapy drugs or other treatments to improve the antitumor response [111]. For example, the combination of atezolizumab and bevacizumab has been approved as a first-line systemic treatment for advanced liver cancer patients. Compared with monotherapy, the objective response rate (ORR) has almost doubled, although it remains at approximately 30% [6, 10, 12, 116, 117]. Nonetheless, there is no study focusing specifically on gut microbiome biomarkers in HCC patients treated with a combination of atezolizumab and bevacizumab, to the best of our knowledge. It is worth noting that as combination immunotherapy’s effectiveness increases, so do the frequency and severity of irAEs [31]. Therefore, using the gut microbiome as a biomarker to predict irAEs seems particularly important in combination immunotherapy treatment.

### New methods and standard

Currently, the most commonly used sequencing methods for gut microbiome are 16S rRNA-seq and metagenomics. To gain more information about the gut microbiome, the metagenomics approach is recommended over 16S rRNA-seq when there are sufficient funds. Multi-omics methods (such as transcriptomics, proteomics, and metabolomics) can also provide additional functional information to clarify the function states of the gut microbiome. The development of new methods is also important. For example, imaging the microbiome may offer unique information that can act as a marker to predict clinical outcomes. It has been observed that patients with advanced NSCLC who received ICIs exhibited better outcomes and higher gut microbiome diversity when PET/CT colon physiologic 18F-FDG uptake was lower [118]. The information obtained from the gut microbiome is vast and complex, hence proper prediction models are equally vital as detection methods. New analysis methods such as artificial intelligence are displaying significant power in analyzing the relationship between the gut microbiome and immunotherapy [54, 75]. Fang et al. established a prediction model trained by random forest using metagenomic sequencing data in NSCLC patients to predict whether a given patient would benefit from ICIs [58]. Additionally, most studies still use RECIST version 1.1 to distinguish responders and non-responders (Tables 1, 2), which may not be suitable for immunotherapy response assessment due to the pseudoprogression. Instead, the iRECIST may be recommended in future clinical studies. Lastly, standardization of the methods is crucial for achieving consistent results across trials, encompassing standardizing the sequencing and analysis techniques, database utilization, and response assessment criteria.

### Multimodal model

**Fig. 4:**
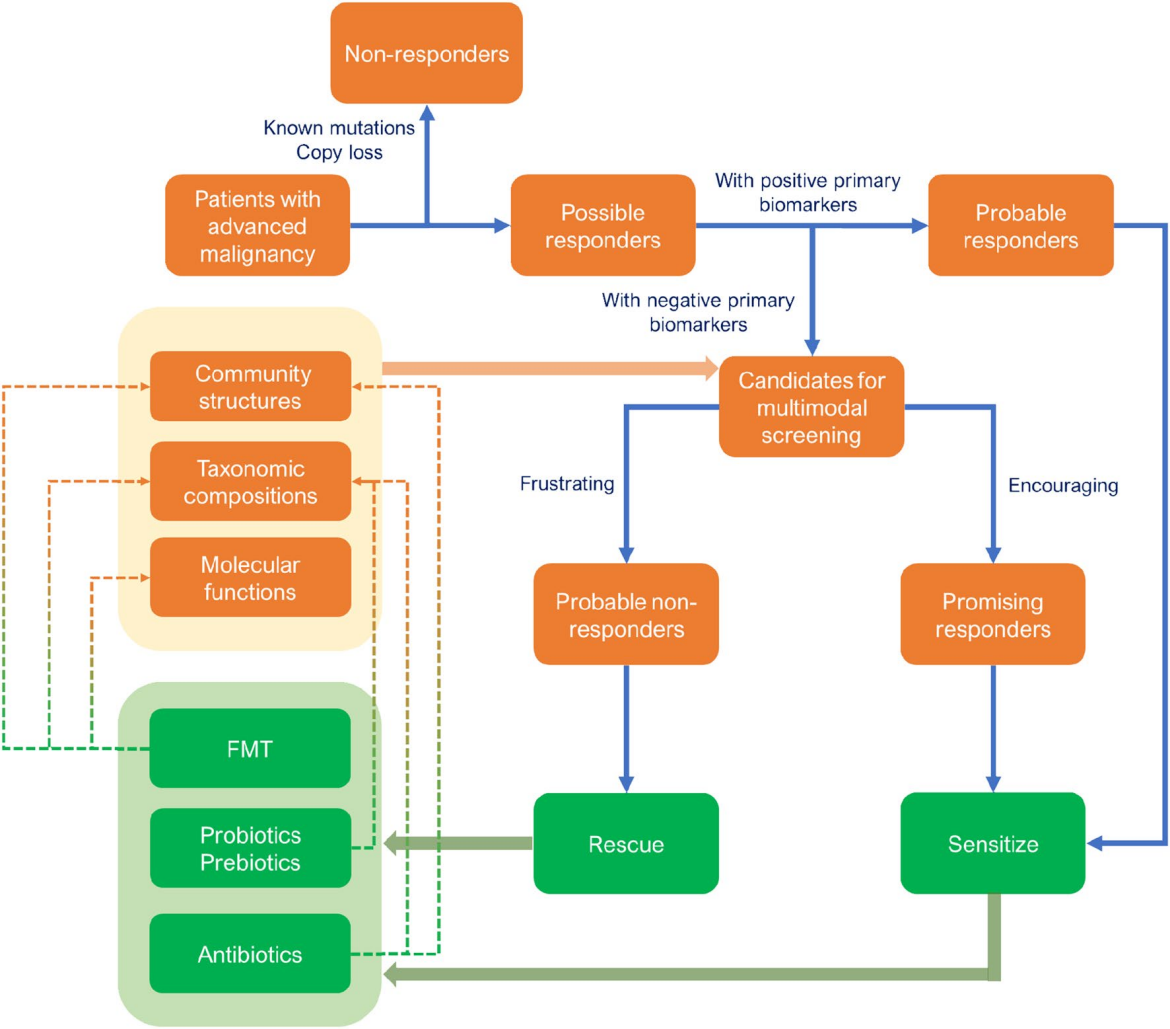
Improved prediction and enhancement process of immunotherapy. To enhance the accuracy of immunotherapy prediction, a combination of various markers, including the gut microbiome, is recommended. Firstly, patients who are most likely to benefit from immunotherapy should be identified while those with specific mutations that hinder its efficacy should be excluded. Secondly, a multiparameter model can be utilized to predict response rates in remaining patients. Manipulation of gut microbiota may serve as a potential intervention to rescue or further enhance treatment outcomes for both non-responders and responders, respectively

Due to the intricacy of host-tumor immunological interactions, a single biomarker may not be sufficient to indicate the most appropriate course of treatment [119]. Instead, a combination of biomarkers may be required for a more accurate prediction of the efficacy of immunotherapy. Vanguri et al. established a multimodal model using a machine-learning approach that integrates radiological, histopathologic, and genomic characteristics. This model showed superior predictive capacity than single-modal measures in patients with NSCLC [120]. In another study involving 7187 patients across 21 cancer types, 36 variables associated with anti-PD-1/PD-L1 therapy response were systematically assessed, and the top three most predictive response factors were identified, including CD8^+^ T-cell abundance (Spearman R = 0.72; p < 2.3 × 10^–4^), TMB (Spearman R = 0.68; p < 6.2 × 10^–4^), and the fraction of samples with high PD-1 gene expression (Spearman R = 0.68; p < 6.9 × 10^–4^) [121]. The combination of these three variables improved prediction accuracy (Spearman R = 0.90; p < 4.1 × 10^–8^) and can explain over 80% of the variance of ORR among different cancer types [121]. Despite its success, there have been no combination biomarkers involving gut microbiome. We proposed an improved prediction and enhancement process that can take full advantage and maximize the impact of different markers (Fig. 4). Firstly, exclude patients with specific mutations, such as JAK1/2 mutations or PD-L1 copy number loss, which may block the effect of immunotherapy [122, 123]. Secondly, select patients who are likely to benefit from immunotherapy, such as those with a high-level expression of PD-L1 in certain types of cancers or having a high relative abundance of specific favorable bacteria. Thirdly, a multiparameter model that integrates gut microbiome biomarkers can be used to predict treatment responses for the remaining patients. For patients who are predicted to have no response, manipulation of the gut microbiota by specific bacteria strain supplement, FMT, or antibiotics usage can be employed to improve the response rate, after which immunotherapy will also be recommended. Patients who were predicted to respond to immunotherapy may also be suitable for gut microbiome manipulation to further enhance the efficacy of immunotherapy. After this procedure, only patients with gene defects who cannot benefit from immunotherapy will be excluded. Finally, while the relationship between antibiotics and immunotherapies has been observed, most of the available data came from studies based on clinical information, and the related studies are only in the initial stage. Thus, to better evaluate the use of antibiotics in immunotherapy, additional experimental data are necessary to elucidate the mechanisms and theories behind them in the future.

## Conclusions

In order to optimize the application of immunotherapy in cancer treatment, it is necessary to identify suitable patients via biomarkers and enhance the efficacy of immunotherapy through various methods. As discussed above, gut microbiota plays an important role in both aspects. This review outlines the predictive features of the gut microbiome, which include community structure, taxonomic compositions, and functional factors. It is also important to note that the characteristics of the gut microbiome as immunotherapy biomarkers can also be modified using manipulation techniques such as increasing favorable microbes (e.g., probiotics/prebiotics supplement), reducing harmful microbes (e.g., selective antibiotics usage), and altering the entire gut microbiome (e.g., FMT or broad-spectrum antibiotics usage). Additionally, when studying the impact of gut microbiome on predicting and enhancing immunotherapy response, it is essential to consider the influence of other factors such as tumor and host factors [65]. Lastly, we proposed several future directions for the application of gut bacteria in immunotherapy, including (i) larger sample size, (ii) new and standardized methods, (iii) more precise and individualized designs, (iv) combined use of different biomarkers, and (v) more scientific experiments. In conclusion, despite ongoing challenges, the potential for clinical use of gut microbiota in immunotherapy is considerable.

## Data Availability

Not applicable.

## Abbreviations

APC: Antigen-presenting cell
BTC: Biliary tract cancer
CAR: Chimeric antigen receptor
CIC: Checkpoint inhibitor colitis
CR: Complete response
CRC: Colorectal cancer
DCs: Dendritic cells
EIOP: Early immunotherapy period
ESCC: Esophageal squamous cell carcinoma
FMT: Fecal microbiota transplantation
GI: Gastrointestinal
GPs: Ginseng polysaccharides
HCC: Hepatocellular carcinoma
HR: Hazard ratio
ICANS: Immune effector cell-associated neurotoxicity syndrome
ICIs: Immune checkpoint inhibitors
irAEs: Immune-related adverse events
mAb: Monoclonal antibody
MDSCs: Myeloid-derived suppressor cells
NGPs: Next-generation probiotics
NHL: Non-Hodgkin lymphoma
NK: Natural killer
NSCLC: Non-small-cell lung cancer
ORR: Objective response rate
ORs: Objective responses
OS: Overall survival
PD: Progressive disease
PD-1: Programmed cell death protein 1
PD-L1: Programmed cell death ligand 1
PFS: Progression-free survival
PR: Partial response
RCC: Renal cell carcinoma
RECIST: Response Evaluation Criteria in Solid Tumors
SCFAs: Short-chain fatty acids
SD: Stable disease
Treg: Regulatory T cell
TKI: Tyrosine kinase inhibitor
TMB: Tumor mutational burden
TNBC: Triple negative breast cancer
UCA: Ursocholic acid
UDCA: Ursodeoxycholic acid
uHCC: Unresectable hepatocellular carcinoma
16S rRNA-seq: 16-Seq ribosomal RNA gene sequencing
